# Supplementary material for: Use of a Novel Passive E-Nose to Monitor Fermentable Prebiotic Fiber Consumption
Source: Sensors (Basel). 2025 Jan 28;25(3):797. doi: 10.3390/s25030797 (PMC11819772; doi:10.3390/s25030797)
Supplement: Supplementary file 1 [file sensors-25-00797-s001.zip › sensors-3422502-supplementary.pdf]

Supplementary Materials

Supplementary Figures

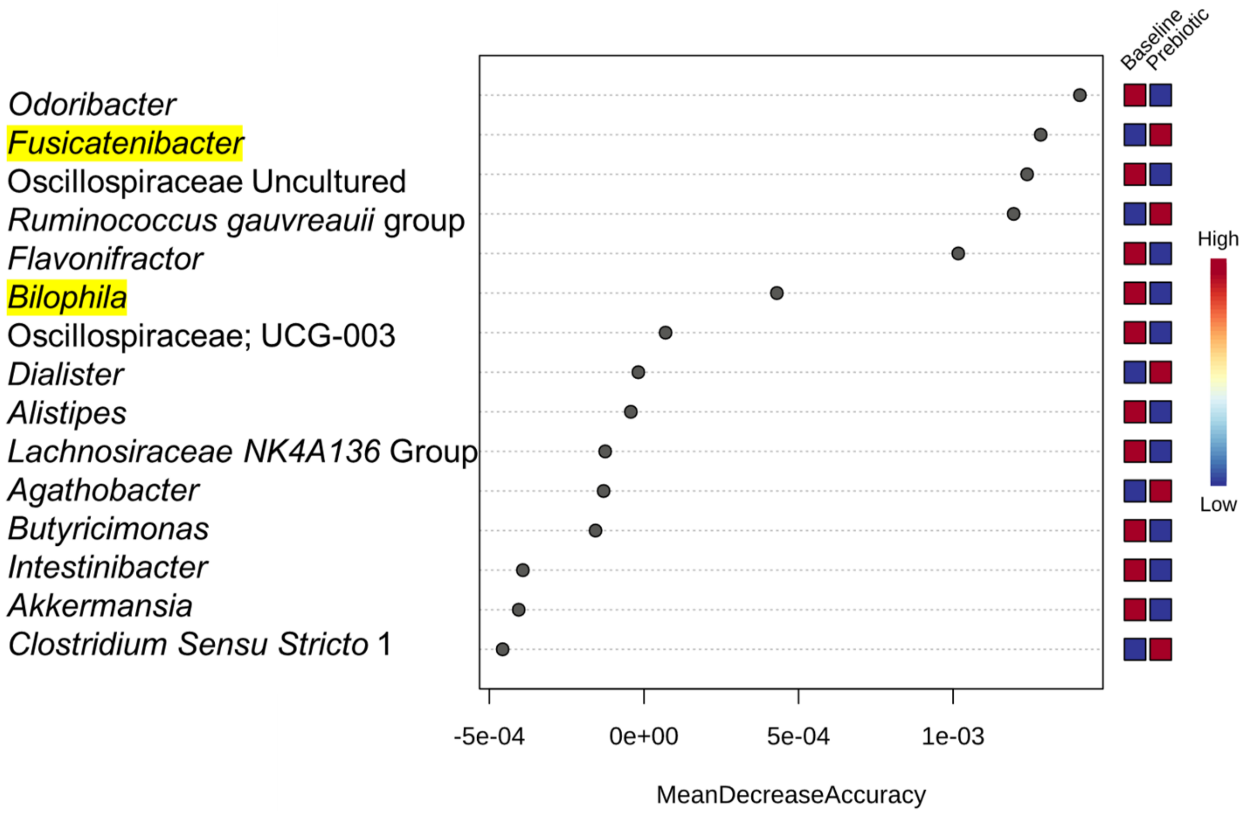

Supplementary Figure S1. Machine Learning Results. Signatures of featured genera of importance between subject's baseline and after prebiotic intervention fecal samples were identified using machine learning algorithm *Boruta*. Genera that encompassed 90% of the overall microbial composition were included in the analysis.

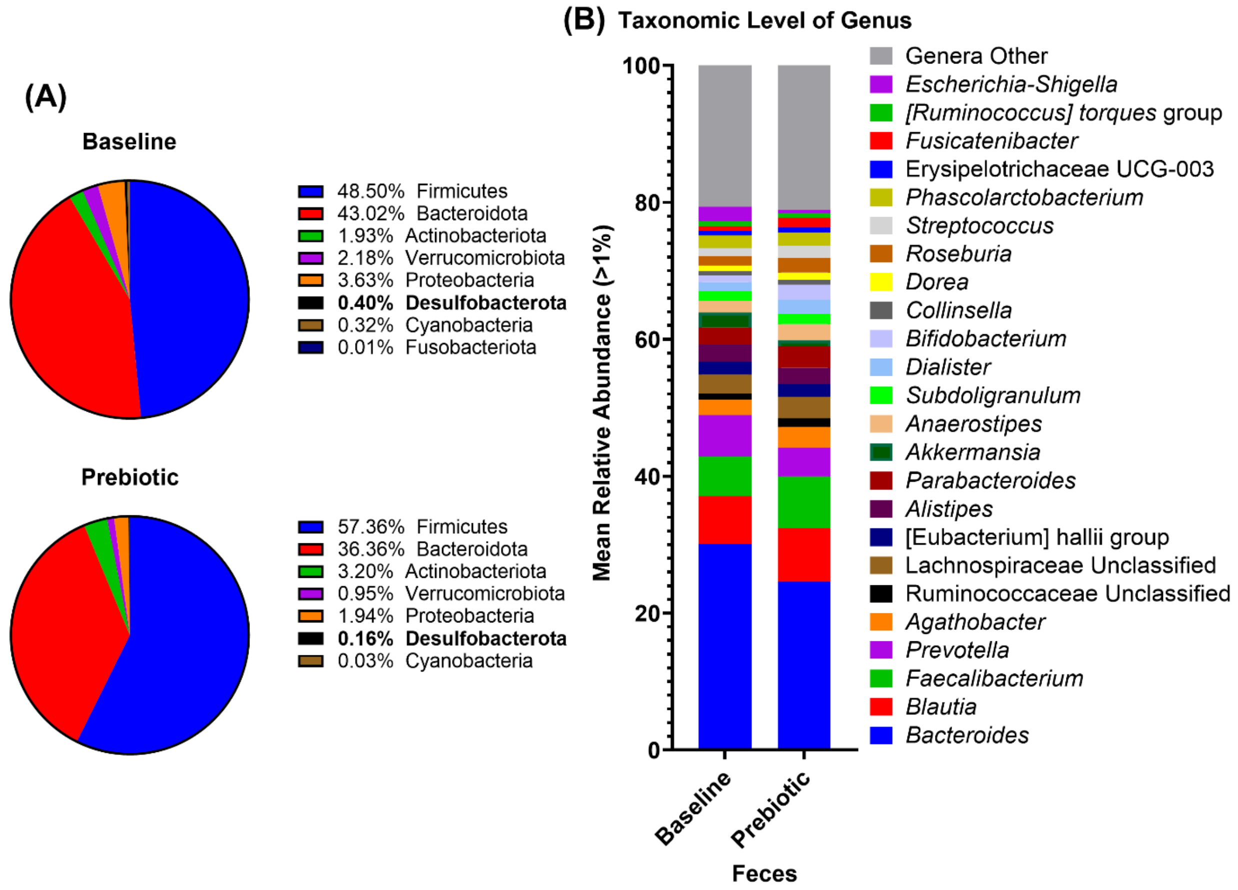

Supplementary Figure S2. Fecal microbial profiles at both the phylum and genus taxonomic levels. Visual comparisons of baseline and after prebiotic intervention fecal samples at the taxonomic level of (**A**) phylum and (**B**) genus. The mean relative abundance of microbial phylum and genera (>1%) are shown. n = 11 subjects per group. The microbiota data are provided in **Supplementary Data File S1**.

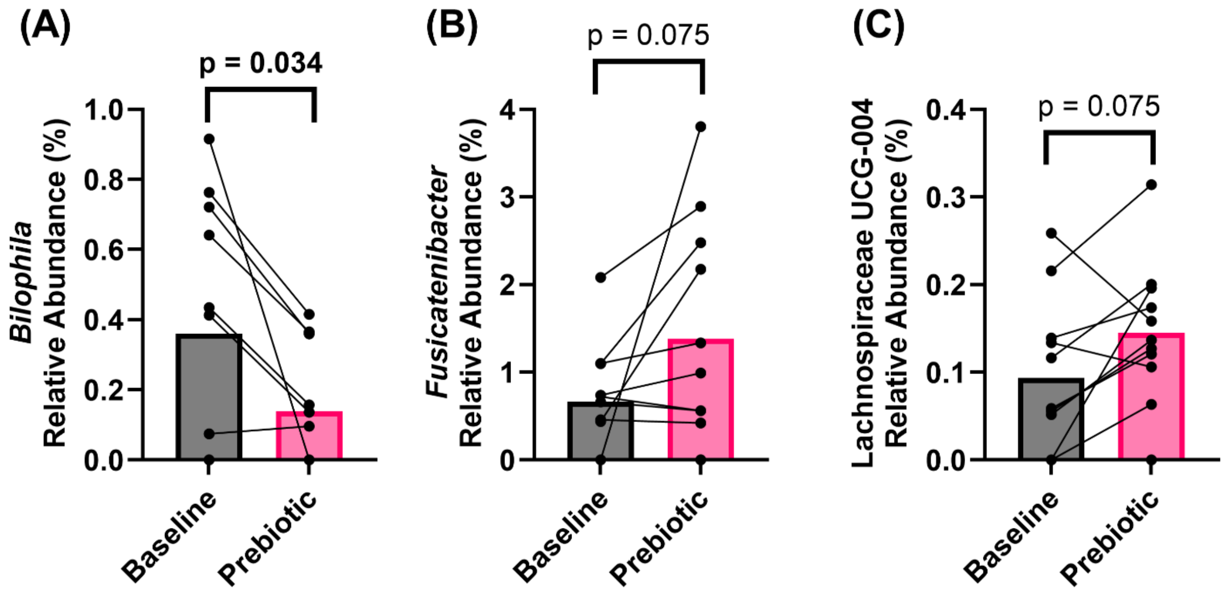

Supplementary Figure S3. Bacterial genera with differential abundance as key features after prebiotic bar consumption. The prebiotic intervention: (**A**) significantly decreased the relative abundance of putative pro-inflammatory genus *Bilophila*; (**B-C**) trending increase for the relative abundances of putative beneficial SCFA-producing bacterial genera *Fusicatenibacter* and Lachnospiraceae UCG-004. Statistical analyses: A line connects each subject at baseline and after the prebiotic intervention. Bar height represents the group's mean value and individual samples are indicated. n = 11. Wilcoxon signed-rank paired test. The microbiota data are provided in **Supplementary Data File S1**.

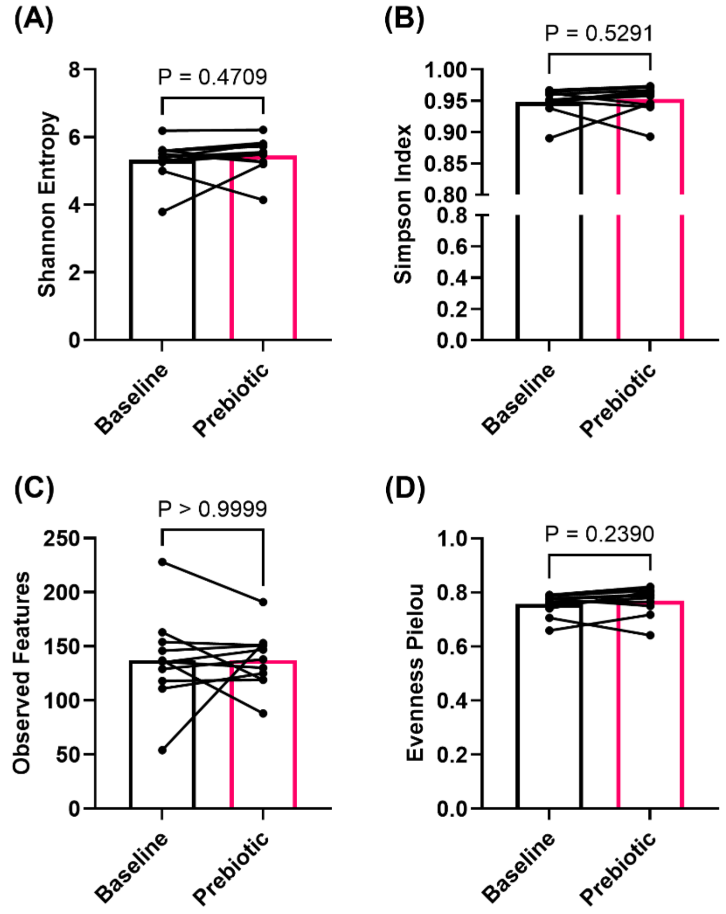

Supplementary Figure S4. Fecal alpha-diversity metrics. Comparison of four alpha-diversity indices at the feature level between baseline and after the prebiotic intervention indicated no significant differences for: (**A**) Shannon Entropy, (**B**) Simpson Index, (**C**) Observed Features, and (**D**) Evenness Pielou. Rarefaction was 16,500 sequences per sample. Statistical analyses: A line connects each subject at baseline and after the prebiotic intervention. Bar height represents the group's mean value and individual samples are indicated. n = 11. Wilcoxon signed-rank paired test.

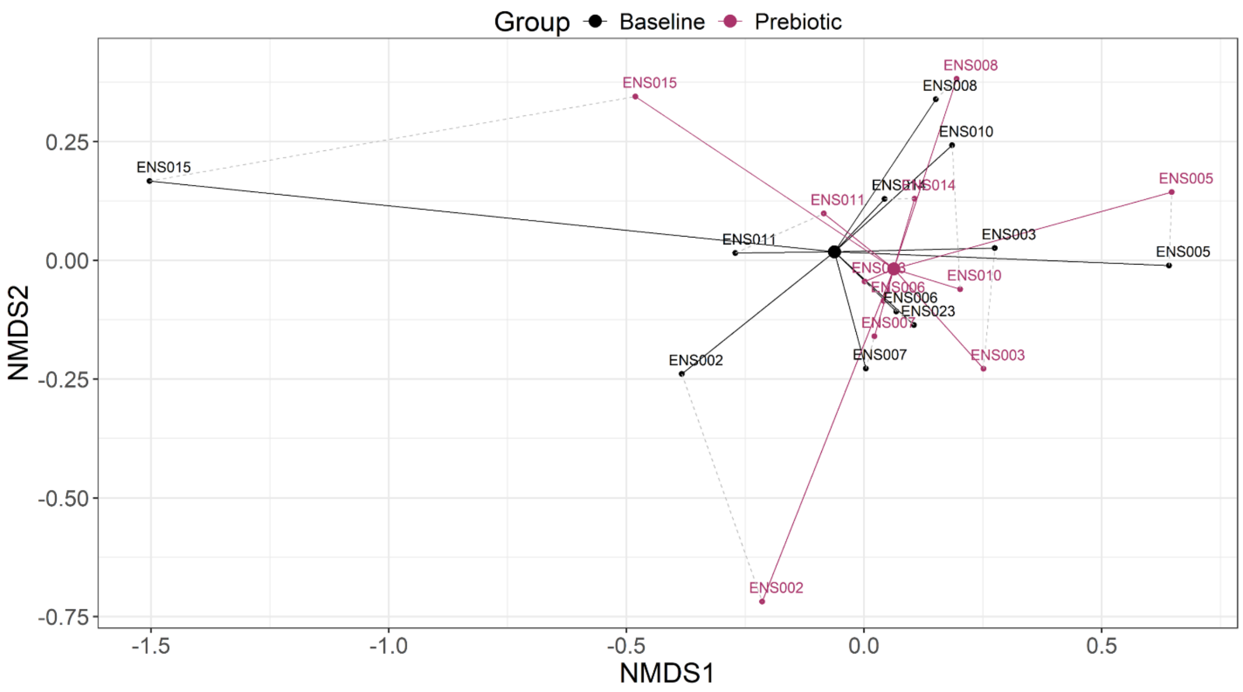

Supplementary Figure S5. Visualization of microbial community structure at baseline and after prebiotic intervention. The overall microbial communities were not significantly different between baseline and after prebiotic consumptions as assessed through PERMANOVA: (p = 0.9081) and PERMDISP (p = 0.995). Microbial community structure was assessed by centroid nonmetric multidimensional scaling (NMDS) at the taxonomic level of genus (n = 11 biologically independent samples assessed at baseline and after the prebiotic intervention). Symbols representing each participant were connected to a centroid representing the mean value of each group: baseline (black) or after prebiotic intervention (pink). A dotted line connects each subject at baseline and after the prebiotic intervention.

Supplementary Methods

Amplicon Sequence Variants

Eight amplicon sequence variants (ASVs) contaminants were identified by *decontam*, and then removed from the dataset prior to downstream analysis of microbial community structure:

d_Bacteria;p_Proteobacteria;c_Alphaproteobacteria;o_Sphingomonadales;f_Sphingomonadaceae;g_Sphingomonas,

d_Bacteria;p_Firmicutes;c_Bacilli;o_Mycoplasmatales;f_Mycoplasmataceae;g_Mycoplasma;s_Mycoplasma_wenyonii,

d_Bacteria;p_Firmicutes;c_Bacilli;o_Mycoplasmatales;f_Mycoplasmataceae;g_Mycoplasma;s_Mycoplasma_wenyonii,

d_Bacteria;p_Proteobacteria;c_Alphaproteobacteria;o_Rhizobiales;f_Xanthobacteraceae;g_Bradyrhizobium,

d_Bacteria;p_Firmicutes;c_Bacilli;o_Mycoplasmatales;f_Mycoplasmataceae;g_Mycoplasma,

d_Bacteria;p_Proteobacteria;c_Gammaproteobacteria;o_Burkholderiales;f_Sutterellaceae;g_Sutterella;s_gut_metagenome,

d_Bacteria;p_Bacteroidota;c_Bacteroidia;o_Bacteroidales;f_Barnesiellaceae;g_Barnesiella,

d_Bacteria;p_Firmicutes;c_Bacilli;o_Lactobacillales;f_Streptococcaceae;g_Streptococcus.
